# Cumulative Family Risk and Cyberbullying Among Chinese Adolescents: The Chain Mediating Role of School Connectedness and Cyber Victimization

**DOI:** 10.3389/fpubh.2022.898362

**Published:** 2022-06-27

**Authors:** Xiong Gan, Hao Li, Guo-xing Xiang, Xin-hua Lai, Xin Jin, Pin-yi Wang, Cong-shu Zhu

**Affiliations:** ^1^Department of Psychology, College of Education and Sports Sciences, Yangtze University, Jingzhou, China; ^2^Department of Psychology, College of Education and Sports Sciences, Yangtze University College of Technology and Engineering, Jingzhou, China

**Keywords:** cumulative family risk, cyberbullying, school connectedness, cyber victimization, adolescence

## Abstract

Abundant evidence has demonstrated that cumulative family risk is associated with cyberbullying. However, few studies to date have investigated how cumulative family risk links to cyberbullying. To fill in these gaps, the present study examined the mediating role of school connectedness and cyber victimization in the relation between cumulative family risk and cyberbullying. A sample of 1,804 Chinese adolescents was recruited to complete measures of cumulative family risk, cyberbullying, school connectedness, cyber victimization, and demographic variables through convenience sampling. There were 813 boys and 991 girls, aged from 13 to 18, with an average age of 16 years (SD = 1.71). Correlational analyses and SPSS macro PROCESS (Model 6) were used for major data analysis. Results indicated that cumulative family risk was positively associated with cyberbullying, and this link could be mediated by school connectedness and cyber victimization. The present study identifies the potential underlying mechanism by which cumulative family risk is associated with adolescent cyberbullying, which has important implications for theory and prevention.

## Introduction

The popularization and development of information technology have created new ways for teenagers to communicate and interact. However, at the same time, it has also led to many practical issues that have spread to the network environment. Offline attacks, for example, extend to the network and turn into cyberbullying (1). Cyberbullying refers to the deliberate and repeated misuse of communication technology by an individual or group to threaten or harm others (2). And it has many manifestations, such as online harassment, cyber threats, online defamation, cyber ostracism, etc. (3, 4). However, because of the concealment of the network, the harm of cyberbullying is easily underestimated. In fact, a large number of empirical studies reveal that cyberbullying has become a common problem behavior among teenagers in China (5–7). On the one hand, the peak of the incidence of cyberbullying is mainly concentrated in adolescence (8). On the other hand, the incidence of cyberbullying is rising day by day. Studies have discovered that 88.72% of teenagers who use the Internet have experienced cyberbullying in China in the past year (9).

Cyberbullying, as a negative form of interpersonal interaction, will harm the cognitive, emotional, and behavioral development of all those involved (perpetrators and victims) (10, 11). Individuals who have been exposed to cyberbullying for a long time may have negative outcomes such as anxiety, depression, sleep difficulties, social impairments, declining academic achievement, absenteeism, and dropping out of school, and more serious individuals may even hurt themselves or others (5, 12–16). For teenagers who carry out bullying, their life satisfaction and academic achievement are lower than those of ordinary teenagers. They are also accompanied by a high level of internalization issues such as depression, anxiety, and loneliness, as well as externalization issues such as smoking, alcohol abuse, and substance misuse (11, 17). Therefore, it is critical to investigate the effective predictors and mechanisms of adolescent cyberbullying.

### The Association Between Cumulative Family Risk and Cyberbullying

There are a variety of reasons why teenagers resort to cyberbullying, and social ecology has been shown to be an effective conceptual framework for understanding traditional bullying (18). Ecological systems theory indicates that individuals' development is affected by many behavioral systems, among which family and school are micro-systems closely related to individuals (19). Families are an important social environment for adolescent. An important feature of this complex social background is the accumulation of potential family risk factors, which may lead to adaptation problems in adolescents.

Risk is considered to be a situation faced by adolescents in a complex social environment that increases the possibility of individual problems in physical and psychological development (20). Family risk refers to the various risk factors faced by individuals in the family system (19). A large number of studies have demonstrated that a single family risk factor can predict adolescent problem behavior. For example, adolescents with lower family economic status have a higher incidence of internalization and externalization problems (21). Frequent parental conflict in the family is an important variable leading to adolescent aggressive behavior (22). Adolescents with poor parent-child relationships are more likely to experience cyberbullying (23). However, in real life, people often have to face a series of risk factors rather than an isolated adverse environment (24).

According to the cumulative risk model, the adverse factors in the environment do not affect adolescents alone but endanger the physical and mental development of adolescents in a superimposed way (25). Previous empirical studies have discovered that cumulative risk factors are positively correlated with Internet addiction and suicide in adolescents (26–29). Therefore, we select five risk factors as cumulative family risk indicators: family economic status, parental relationship, parent-child relationship, family structure, and parental educational level. And we propose that cumulative family risk may be positively associated with cyberbullying (Hypothesis 1).

### School Connectedness as a Mediator

As one of the important variables in the ecological microsystem, school connectedness is defined as “students' perceptions that adults care about their learning and about them as individuals” (30). Many studies have shown that single family risk factors (such as parent-child relationship, parental educational level) are negatively correlated with adolescent school connectedness (31). Adolescents affected by family risks are prone to problem behaviors such as aggression, unsociability, and poor social skills, which will affect them to establish good interpersonal relationships with teachers and classmates at school (32). A recent empirical study also found that poor children exposed to cumulative family risk tend to show lower school connectedness (33).

On the other hand, some research has indicated that adolescents with higher school connectedness tend to show less cyberbullying (10). Social control theory also reveal that students with higher school connectedness will actively internalize the school's goals, expectations, and values to reduce problem behavior (34). That means cumulative family risk can lead to problem behavior (such as cyberbullying) by weakening their school connectedness among adolescents. For example, Li and her colleagues revealed that parent-child attachment can indirectly affect adolescent aggressive behavior through school connectedness (35). Therefore, we hypothesize that school connectedness may act as a mediator in linking cumulative family risk to cyberbullying (Hypothesis 2).

### Cyber Victimization as a Mediator

Many researchers have found that individuals who have suffered cyberbullying are more likely to become cyber bullies (5, 36). Xiao and Wong (36) indicated that 60% of the cyber bullies in their study had experienced the same cyberbullying, and they deemed that cyber victimization can significantly influence cyberbullying. According to the general strain theory, after experiencing stressful events, negative emotions (such as anger, anxiety, depression, and fear) can prompt individuals to adopt negative coping styles (37). The negative emotions caused by cyber victimization, such as depression (16), may promote individuals to adopt non-adaptive or pathological coping styles, such as cyberbullying. Some studies have also indicated that early cyber victimization can significantly predict later cyberbullying (6, 38).

On the other hand, the cyberbullying model indicate that perceived parental support is an important factor affecting adolescent cyber victimization, and cyber victimization is closely related to family risk factors (39). Related studies have also found that the negative experiences of cyber victims (such as various family risk factors) are the main source of their stress (40, 41). A warm parenting style can negatively predict adolescent cyber victimization (39). That means adolescents affected by cumulative family risk may suffer more cyber victimization, which in turn leads to more cyberbullying. Therefore, we hypothesize that cyber victimization may act as a mediator in linking cumulative family risk to cyberbullying (Hypothesis 3).

### The Chain Mediating Effect of School Connectedness and Cyber Victimization

In recent years, research on the relationship between school connectedness and cyberbullying has been increasing. Previous studies have shown that students with higher levels of school connectedness tend to have lower levels of peer victimization (42). Specifically, adolescents who have a close relationship with school generally have a positive interpersonal relationship and get more attention and support from their teachers and students, which reduces the possibility of being bullied (43). Thus, it is reasonable to speculate that Chinese adolescents with high school connectedness are less likely to suffer from cyberbullying. A meta-analysis also reveals that adolescent school connectedness is negatively associated with cyber victimization (44). Therefore, we hypothesize that school connectedness and cyber victimization paly a chain mediating role between cumulative family risk and cyberbullying (Hypothesis 4).

### The Present Study

Although it has been suggested that school connectedness and cyber victimization are related to cumulative family risk and cyberbullying, it remains unclear how school connectedness and cyber victimization influence this relationship. This is the first study, to our knowledge, that takes both the mediating effects of school connectedness and cyber victimization into consideration. This research will contribute to a better understanding of the mechanisms that link cumulative family risk and cyberbullying, as well as to the advancement of ecological systems theory, social control theory, and general strain theory in the field of cyberbullying.

**Figure 1:**
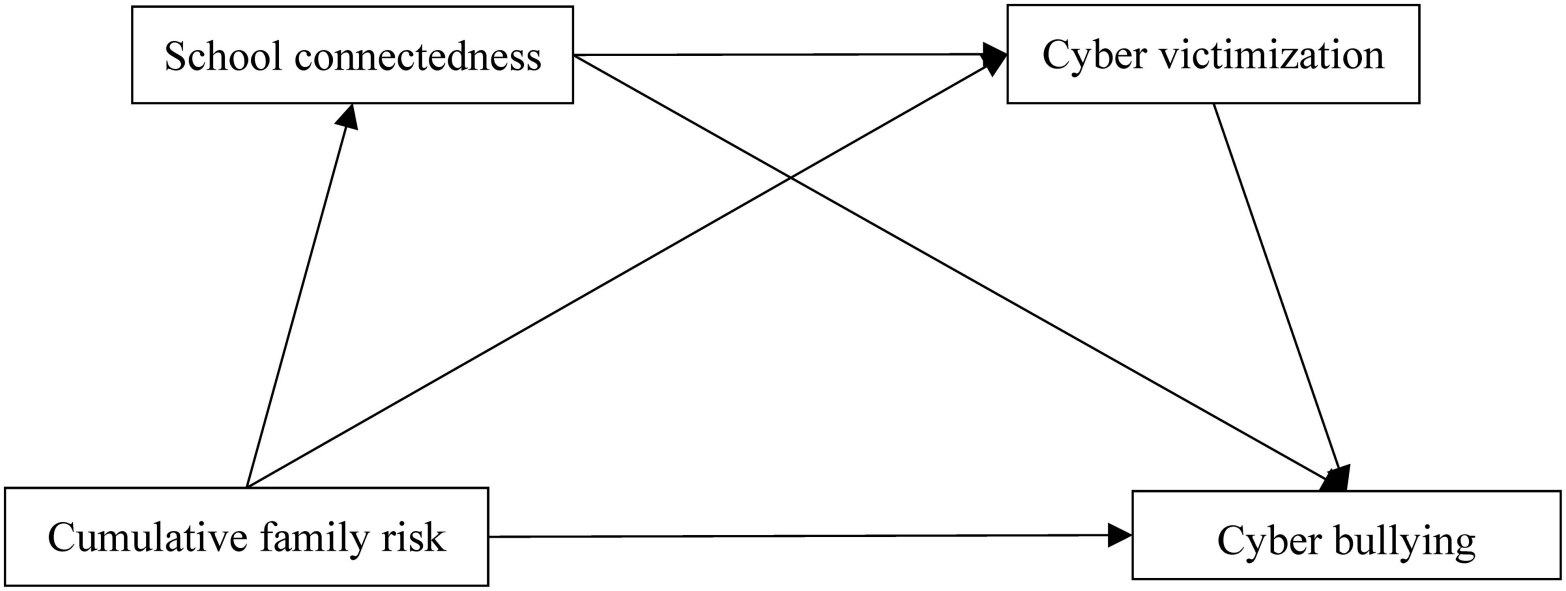
Hypothesized relationships among cumulative family risk, school connectedness, cyber victimization, and cyber bullying.

In summary, the present study tested the mediating effects of school connectedness and cyber victimization on the relationship between cumulative family risk and cyberbullying by using a sample of Chinese adolescents. Based on previous empirical research, ecological systems theory, social control theory, and general strain theory, we proposed four hypotheses: (1) cumulative family risk may be positively associated with cyberbullying; (2) school connectedness may act as a mediator in linking cumulative family risk to cyberbullying; (3) cyber victimization may act as a mediator in linking cumulative family risk to cyberbullying; (4) school connectedness and cyber victimization paly a chain mediating role between cumulative family risk and cyberbullying. The hypothetical model of the study is shown in Figure 1.

## Methods

### Participants

The participants in this study were recruited from two middle schools in Hubei and Guizhou, China through random cluster sampling. A total of 2,000 questionnaires were distributed, of which 1,804 were valid, and the response rate was 90.2%. There were 813 boys and 991 girls, aged from 13 to 18, with an average age of 16 years (SD = 1.71). Moreover, the eligible participants were selected based on the following criteria: (1) participants who were adolescents, (2) adolescents who received consent from their guardians to participate, and (3) adolescents who agreed to participate.

### Procedures

The present study was approved by the Research Ethics Committee of the College of Education and Sports Sciences, Yangtze University. Convenience sampling was adopted to choose six to seven classes in each grade. Participants and their parents or legal guardians were provided with written consent forms, which informed them that personal information would be kept confidential and their responses would be used only for research purposes. The data was collected by trained senior students majoring in psychology during class time. To encourage honest reporting, adolescents were given approximately 30 min to complete the anonymous questionnaires.

### Measures

#### Cumulative Family Risk

The study selected five family risk factors for measurement based on relevant studies in the field of cumulative risk: family economic status, parental relationship, parent-child relationship, family structure, and parental educational level (45, 46). Among them, family economic status, parent-child relationship and parental relationship are continuous variables. In this study, the family risk factors in the cumulative risk model are dichotomized (0 and 1), and the 75th or 25th percentile is used as the classification standard for risk factors in continuous variables. And a code of 1 indicates that there is a risk, whereas a code of 0 indicates that there is no risk. Finally, the score of each family risk factor after coding is added to get the cumulative family risk index. The score is between 0 and 5. And the higher the score, the higher the level of cumulative family risk. Cronbach's alpha for cumulative family risk scale in the present study was 0.61.

##### Family Economic Status

Family economic status was measured by the Chinese version of the family financial difficulty scale (21), which was revised from the original version developed by (47). A sample item was “My family doesn't have enough money to buy my favorite food.” Adolescents were asked to answer four items on a 5-point scale ranging from 1 (never) to 5 (always). The total score was calculated for each participant, with higher scores indicating more difficulty with family finances. Cronbach's alpha for this scale in the present study was 0.75. If the total score of the participants is higher than or equal to the 75th percentile, the code is 1, and *vice versa*.

##### Parental Relationship

Referring to previous studies (46), two items were used to measure the parental relationship. The two items are “Is there a good relationship between your father and mother?” and “Do your parents often quarrel?”, respectively. Participants were asked to respond to two items on a five-point Likert-type scale ranging from 1 (very bad/never) to 5 (very good/always), with higher mean scores indicating a better parental relationship. If the total score of the participants is ≤ 25th percentile, the code is 1, and *vice versa*.

##### Parent-Child Relationship

Parent-child relationship was measured by the Chinese version of the parental-child relationship scale (21), which was revised from the original version developed by Furman and Buhrmester (48). A sample item was “Do your parents like or praise what you do?”. Adolescents were asked to answer seven items on a 5-point scale ranging from 1 (never) to 5 (always). The mean score was calculated for each participant, with higher scores indicating the better parent-child relationship. Cronbach's alpha for this scale in the present study was 0.66. If the total score of the participants is ≤ 25th percentile, the code is 1, and *vice versa*.

##### Family Structure

Referring to previous studies (49), one item was used to measure the family structure. That is “Who are the families you live with now?”. Participants living with the “biological parents” were regarded as risk-free (coded as 0). In other cases, it is encoded as 1, which means that there is a risk.

##### Parental Educational Level

Two items were used to measure the parental educational level. The two items are “How educated is your mother?” and “How educated is your father?”. If the parental educational level is higher than that of a senior high school, the code is 0, and *vice versa*.

#### School Connectedness

School connectedness was measured with the 6-item self-report questionnaires (50). Example items include “I am close to the people in our school.” And all items were rated on a 5-point scale ranging from 1 (disagree) to 5 (agree). Total scores were calculated, with higher scores meaning a greater level of school connectedness. This measure demonstrated good reliability and validity among Chinese adolescents (51). In the present study, the Cronbach's alpha was 0.83.

#### Cyberbullying and Cyber Victimization

Cyberbullying and cyber victimization was measured with the 12-item self-report questionnaire, which was adapted from Cyberbullying/Being Cyber Bullied Questionnaire (52). The first six items measure cyber victimization, and the last 6 items measure cyberbullying. Example items include “Some people used to laugh at me through email, SMS, instant messaging (QQ, Wechat), social networking sites (Qzone, Renren, Wechat moments) and so on.” The participants were asked to report the frequency of cyberbullying/cyber victimization in the last year. All items were rated on a 7-point scale (from 0 = never to 6 = 6 times or more). Total scores were calculated, with higher scores meaning the higher the degree of cyberbullying/cyber victimization. This measure demonstrated good reliability and validity among Chinese adolescents (53). In the present study, the Cronbach's alpha was 0.79.

### Statistical Analysis

Firstly, we conducted descriptive statistics and Pearson correlations to examine the means, standard deviations, and bivariate associations for all variables. Then, we employed the SPSS macro PROCESS (model 6) suggested by Hayes to test the proposed moderated mediation model (54). This SPSS macro has been used to test mediating models in several studies, in which this SPSS macro showed higher statistical testability (55, 56). The missing data were handled with the full information maximum likelihood estimation (FIML).

## Results

### Preliminary Analyses

The distribution of sample gender and age is shown in Table 1. Means, standard deviations, and bivariate associations are shown in Table 2. As can be seen in the table, cumulative family risk was positively correlated with cyberbullying (*r* = 0.105, *p* < 0.001) and cyber victimization (*r* = 0.144, *p* < 0.001) and negatively associated with school connectedness (*r* = −0.216, *p* < 0.001). School connectedness was negatively associated with cyber victimization (*r* = −0.221, *p* < 0.01) and cyberbullying (*r* = −0.192, *p* < 0.01). Cyber victimization was positively correlated with cyberbullying (*r* = 0.521, *p* < 0.001).

**Table 1:** Distribution of sample gender and age.

**Gender/age**	**13**	**14**	**15**	**16**	**17**	**18**
Boy	76	126	103	120	144	244
Girl	74	181	105	136	194	301

*N = 1,084*.

**Table 2:** Descriptive statistics and interrelations among variables.

**Variable**	***M***	**SD**	**1**	**2**	**3**	**4**
1. Cumulative family risk	1.835	1.268	1.000			
2. Cyberbullying	0.640	1.665	0.105***	1.000		
School connectedness	22.095	4.052	−0.216***	0.192**	1.000	
4. Cyber victimization	2.050	3.748	0.144***	0.521***	0.221**	1.000

**p < 0.01.

***p < 0.001.

### The Chain Mediating Effects Analyses

Hayes's SPSS macro PROCESS was adopted to examine the proposed mediation model. Table 3 presented the main results after controlling adolescent gender. Cumulative family risk positively predicted cyberbullying in equation 1 (β = 0.054, *p* < 0.001). However, after putting the intermediary variables into the model, the direct effect is no longer significant in equation 4 (β = 0.009, *p* > 0.05). And Cumulative family risk negatively predicted school connectedness in equation 2 (β = −0.205, *p* < 0.001) and positively predicted cyber victimization in equation 3 (β = 0.077, *p* < 0.001). School connectedness negatively predicted cyber victimization in equation 3 (β = −0.158, *p* < 0.001) and cyberbullying in equation 4 (β = −0.043, *p* < 0.001). Cyber victimization positively predicted cyberbullying in equation 4 (β = 0.328, *p* < 0.001).

**Table 3:** Regression results for the conditional indirect effects.

**Predictor variable**	**Outcome variable**	***R***	***R^2^***	***f^2^***	**β**	***t***	**Boot LLCI**	**Boot ULCI**
**Equation 1**
Gender	Cyberbullying	0.187	0.035		−0.113	−4.953***	−0.158	−0.068
CFR					0.054	4.623***	0.031	0.076
**Equation 2**
Gender	SC	0.216	0.047		−0.022	−0.500	−0.106	0.063
CFR				0.049	−0.205	−9.355***	−0.248	−0.162
**Equation 3**
Gender	CV	0.291	0.085		−0.120	−3.619***	−0.185	−0.055
CFR				0.021	0.077	4.466***	0.043	0.111
SC				0.070	−0.158	−8.703***	−0.194	−0.122
**Equation 4**
Gender	Cyberbullying	0.534	0.285		−0.076	−3.837***	−0.114	−0.0347
CFR				0.011	0.009	0.867	−0.011	0.029
SC				0.031	−0.043	−3.908***	−0.064	−0.021
CV				0.341	0.328	23.499***	0.301	0.356

*CFR, cumulative family risk; SC, school connectedness; CV, cyber victimization; LL, low limit; CI, confidence interval; UL, upper limit*.

*Unstandardized regression coefficients are reported. Bootstrap sample size = 5,000*.

**p < 0.01.

***p < 0.001.

**Figure 2:**
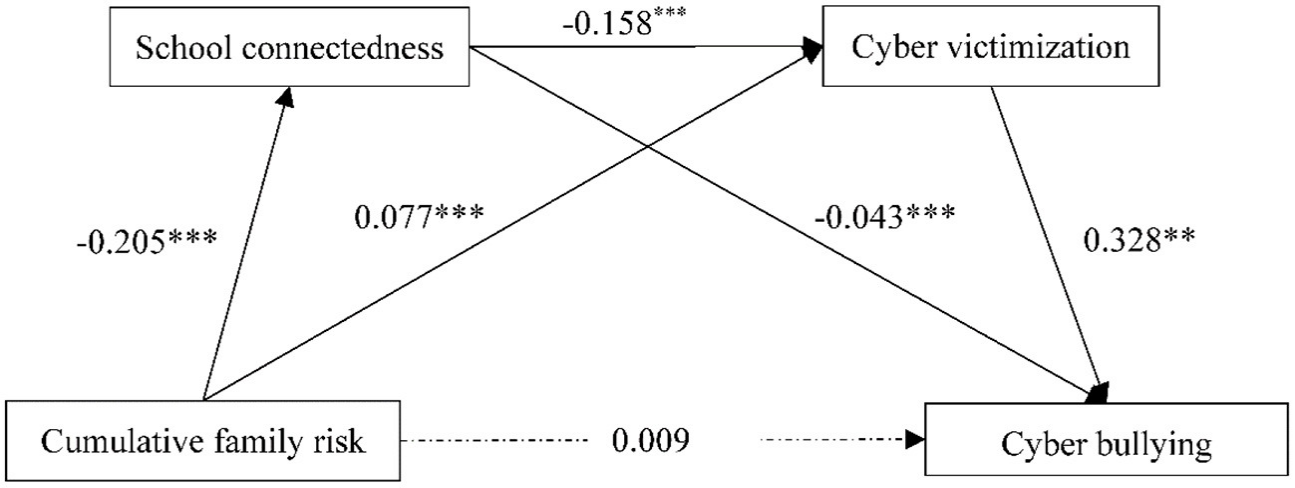
The chain mediating effect of school connectedness and cyber victimization. **p < 0.01, ***p < 0.001.

The results of the chain mediating effect of school connectedness and cyber victimization are shown in Table 4 and Figure 2. We found that the total indirect effect was 0.045, which accounted for 83.333% of the total effect (0.054) in the relationship between cumulative risk family and cyberbullying. Specifically, the total indirect effect included three different pathways. Cumulative family risk affected adolescent cyberbullying through the mediating role of school connectedness, through the mediating role of cyber victimization, and through the chain mediating role of both school connectedness and cyber victimization, which were shown in the indirect effects 1, 2, and 3, respectively. Furthermore, indirect effects 1, 2, and 3 accounted for 16.667, 46.296, and 20.370% of total effect, respectively. And the all 95% confidence intervals did not overlap with zero, which indicated that all indirect effects were significant.

**Table 4:** Indirect effect of school connectedness and cyber victimization.

	**Effect**	**Boot SE**	**Boot LLCI**	**Boot ULCI**	**Ratio of indirect to** **total effect**
Total indirect effect	0.045	0.007	0.031	0.060	83.333%
Indirect effect 1	0.009	0.003	0.004	0.015	16.667%
Indirect effect 2	0.025	0.006	0.014	0.038	46.296%
Indirect effect 3	0.011	0.002	0.007	0.015	20.370%

*Indirect effect 1 was cumulative family risk → school connectedness → cyberbullying. Indirect effect 2 was cumulative family risk → cyber victimization → cyberbullying. Indirect effect 3 was cumulative family risk → school connectedness → cyber victimization → cyberbullying. Bootstrap sample size = 5,000*.

*LL, low limit; CI, confidence interval; UL, upper limit*.

## Discussion

Although some empirical has showed that the positive association between cumulative family risk and adolescent cyberbullying, the mediating mechanisms underlying this relation had not been identified. According to ecological system theory, cumulative risk model, and empirical research, the current study examined the mediation effect of school connectedness and cyber victimization on the relationship between cumulative family risk and cyberbullying. Findings revealed that cumulative family risk has a positive effect on adolescent cyberbullying, and school connectedness and cyber victimization paly a chain mediating role between cumulative family risk and cyberbullying. These observations expand understanding of the complex relations between cumulative family risk and cyberbullying among teenagers in China, and provide reference suggestions for the prevention and intervention of cyberbullying. However, it is worth noting that even if the *p*-value of the regression is significant, some of the corresponding effect sizes only achieve a small effect. Therefore, it is necessary to be careful in both practical interpretation and application.

In line with previous research documenting that cumulative family risk positively predicts cyberbullying among Chinese adolescents (27, 57). Teenagers affected by the negative family environment may socialize offline problem behaviors (such as traditional bullying) as a reasonable way to interact with their peers, and further reflect this way in face-to-face or online communications, which develops into cyberbullying (58). It is worth noting that after putting the intermediary variables into the model, the direct effect between cumulative family risk and cyberbullying is no longer significant. This demonstrates that adolescents experience more family risk, which does not imply that it will result in cyberbullying. In addition, school connectedness and cyberbullying are significant determinants in family cumulative risk and cyberbullying.

The findings reveal that school connectedness act as a mediator in linking cumulative family risk to cyberbullying. Ecosystem theory shows that family and school are not independent of each other, on the contrary, the function of one environment (such as cumulative family risk) will affect the operation of the other environment (school connectedness). Adolescents exposed to fewer family risks may have a more positive connection with school, thus feeling a sense of belonging to the school and deeming them to get more support from teachers and peers (59, 60). On the other hand, social control theory indicates that students who have strong emotional ties to school are more likely to internalize their expectations and values from their attachment individuals (such as teachers and classmates) than those who have less emotional ties to school, thus prompting them to stay away from cyberbullying (34).

The results also show that cyber victimization may act as a mediator in linking cumulative family risk to cyberbullying, which expands the previous research. The negative experiences of cyber victims (such as various family risk factors) are the main source of their stress (61). Adolescents may escape from reality through the Internet, which increases the possibility of being bullied on the Internet. On the other hand, according to the general stress theory, adolescents who are being bullied on the Internet will behave cyberbullying (5, 7). The negative emotions caused by cyber victimization, such as depression, anger, and frustration (62), may promote individuals to adopt non-adaptive or pathological coping styles, such as cyberbullying. Moreover, the multiple identities and false identities provided by the virtual platform also increase the possibility of cyberbullying by cyber victims (40, 63). What needs to be considered is that the effect size of network bullying on cyberbullying is large (64), which coincides with a large number of research results (5, 7, 63, 65). Chinese educators and parents should pay more attention to the online status of teenagers and reduce their transformation from Internet victims to Internet perpetrators.

At last, another result that deserves our attention is that school connectedness and cyber victimization paly a chain mediating role between cumulative family risk and cyberbullying. Adolescents with high cumulative family risk will show fewer school connectedness and then have a higher risk of cyber victimization (66), leading to more cyberbullying. A family is an important social unit for adolescents to live and grow in, and a good family environment is easier to cultivate for adolescents with physical and psychological health (67). On the contrary, teenagers who face more cumulative family risks may have a series of internal and external problems (45). Teenagers may develop personal traits such as inferiority, helplessness, low self-confidence, and shame, which make it difficult for them to feel the support from teachers and classmates in school and to have a positive connection with the school. Even these traits make individuals vulnerable to cyber victimization.

## Limitations and Future Directions

Limitations of this study and future directions should be noted. First, due to our cross-sectional research design, causality cannot be established. Future longitudinal or experimental studies can further examine the causal relationship between cumulative family risk and cyberbullying. Second, cumulative family risk in this study has a low prediction of cyberbullying and cyber victimization, which may be due to the limited risk factors selected. Related studies have demonstrated that other risk factors such as childhood abuse and parental marital relationships can influence adolescent problem behavior (68, 69). Therefore, future research can include more family risk factors to further explore the effect of cumulative family risk on cyberbullying. Third, self-reports may be subject to increased biases (e.g., socially desirable response) and inflated associations between antecedent and outcome variables (70). Future studies should allow for a multidimensional approach to collect more objective. Fourth, some of the regression effect sizes only achieve a small effect. Thus, it is necessary to be careful in both practical interpretation and application. Fifth, the counterbalancing technique should be applied to the order of questionnaires. Hence, more rigorous measurement methods can be used for analysis in the future. Sixth, the total Cronbach's alpha of cumulative family risk in this study is not high, and more rigorous and scientific measurement tools can be selected for future research in order to get more accurate results. Finally, the results of the present study also need to be extended to a more representative sample of Chinese adolescents and to adolescents from other cultural backgrounds for a wider test.

## Conclusion

Taken together, the current study reveals that cumulative family risk was positively associated with cyberbullying, and this link could be mediated by school connectedness and cyber victimization.

## Data Availability Statement

The raw data supporting the conclusions of this article will be made available by the authors, without undue reservation.

## Ethics Statement

The studies involving human participants were reviewed and approved by Research Ethics Committee of College of Education and Sports Sciences, Yangtze University. Written informed consent to participate in this study was provided by the participants' legal guardian/next of kin. Written informed consent was obtained from the individual(s), and minor(s)' legal guardian/next of kin, for the publication of any potentially identifiable images or data included in this article.

## Author Contributions

XG, HL, and X-hL designed the work. X-hL, XJ, and C-sZ collected the data. XG, HL, and G-xX analyzed and drafted the manuscript. XG, HL, and P-yW revised the manuscript. All authors contributed to the article and approved the submitted version.

## Funding

This work was funded by Youth project of Science and Technology Research Plan of Department of Education of Hubei Province in 2020 (Q20201306), Project of Social Science Foundation of Young Scholar Support Plan of Yangtze University in 2020 (2020skq24), and Project of Social Science Foundation of Yangtze University in 2021 (2021csy15).

## Conflict of Interest

The authors declare that the research was conducted in the absence of any commercial or financial relationships that could be construed as a potential conflict of interest.

## Publisher's Note

All claims expressed in this article are solely those of the authors and do not necessarily represent those of their affiliated organizations, or those of the publisher, the editors and the reviewers. Any product that may be evaluated in this article, or claim that may be made by its manufacturer, is not guaranteed or endorsed by the publisher.

## References

[1] Tokunaga RS. Following you home from school: a critical review and synthesis of research on cyberbullying victimization. Comput Human Behav. (2009) 26:277–87. 10.1016/j.chb.2009.11.014

[2] Savage MW Tokunaga RS. Moving toward a theory: testing an integrated model of cyberbullying perpetration, aggression, social skills, and Internet self-efficacy. Comput Human Behav. (2017) 71:353–61. 10.1016/j.chb.2017.02.016

[3] Willard NE. The authority and responsibility of school officials in responding to cyberbullying. J Adolesc Health. (2007) 41:S64–5. 10.1016/j.jadohealth.2007.08.013 18047949

[4] Wójcik M Rzeńca K. Disclosing or hiding bullying victimization: a grounded theory study from former victims' point of view. School Mental Health. (2021) 13:1–11. 10.1007/s12310-021-09447-5

[5] Zhu XW Zhou ZK Chu XW Lei YJ Fan CY. The trajectory from traditional bullying victimization to cyberbullying: a moderated mediation analysis. Chin J Clin Psychol. (2019) 27:492–6. 10.16128/j.cnki.1005-3611.2019.03.013

[6] Shi HF Fan CY Chu XW Zhang XC Wu LL. Cyberbullying victimization and cyberbullying perpetration among adolescents: the roles of belifs about aggression and dual-mode of self-control. J Psychol Sci. (2020) 43:1117–24. 10.16719/j.cnki.1671-6981.20200513

[7] Wang QQ Fan CY Chu XW. The relationship between adolescent cybervictimization and cyberbullying: a moderated model. Psychol Deve Educ. (2020) 36:216–27. 10.16187/j.cnki.issn1001-4918.2020.02.1133658955

[8] Li YJ. Co-occurrence of cyber victimization and traditional victimization in adolescents. Chin J Clin Psychol. (2015) 23:346–9+353. 10.16128/j.cnki.1005-3611.2015.02.036

[9] Chen QY Tang HY Zhang L Zhou ZK. Cyberbullying and its risk factors in Chinese adolescents' usage of social networking sites -based on a survey of 1103 secondary school students. Chin J Special Educ. (2016) 89–96. 10.16128/j.CNKI:SUN:ZDTJ.0.2016-03-014

[10] Fan CY Wang QQ Chu XW Teng YJ. Influence factors, consequence and educational countermeasures of youth internet bullying. Educ Res Exp. (2018) 3:93–6.

[11] Liu ZJ Liang N. Review of the intervention of adolescent's cyberbullying. J School Stud. (2018) 15:87–92+101. 10.3969/j.issn.1005-2232.2018.02.011

[12] Zhou ZK. Cyberbullying and its risk factors among chinese high school students. Sch Psychol Int. (2013) 34:630–47. 10.1177/0143034313479692

[13] Wang YL Zhang MQ Tan GL Lin F. The relationship between cybervictimization and selflnjury of adolescents: the moderating role of friendship quality and ruminative response. J Psychol Sci. (2020) 43:363–70. 10.16719/j.cnki.1671-6981.20200215

[14] Zhang XC Fan CY Chu XW Chu YH. Effect of cyberbullying victimization on adolescents' sleeping problem: the chain mediating role of perceived stress and depression. J Psychol Sci. (2020) 43:378–85. 10.16719/j.cnki.1671-6981.20200217

[15] Li YJ. The co-occurring patterns of bullying/victimization behaviors in adolescents. Chin J Clin Psychol. (2021) 29:489–95+472. 10.16128/j.cnki.1005-3611.2021.03.009

[16] Zhou T Shi HF Fan CY Zheng YL Fang Y. Cybervictimization and depression among children: a modetated mediation model. Chin J Clin Psychol. (2022) 30:72–76. 10.16128/j.cnki.1005-3611.2022.01.015

[17] Feng ZY Wang PY Huang Q Huang XN Xu MJ Yang XG. The relationship between social support, resilience, cyber-bullying and life satisfaction among college students. Chin J Health Educ. (2016) 32:8–11+31.

[18] Espelage DL. Ecological theory: preventing youth bullying, aggression, and victimization. Theory Pract. (2014) 53:257–64. 10.1080/00405841.2014.947216

[19] Bronfenbrenner U. Making human beings human: bioecological perspectives on human development. Br J Dev Psychol. (2004) 23:143–51. 10.1348/026151004X21134

[20] Jessor R. New Perspectives on Adolescent Risk Behavior. Cambridge, MA: Cambridge University Press (1998). 10.1017/CBO9780511571138

[21] Li DP Zhang W. Adolescence's family financial difficulty and social adaptation: coping efficacy of compensatory, mediation, and moderation effects. J Beijing Normal Univ. (2010) 4:22–32. 10.3969/j.issn.1002-0209.2010.04.002

[22] Chen T Zhang Y Ma ZQ. The effect of inter-parental conflict on aggression of junior middle school student: the chain mediating role of regulatory emotional self-efficacy and emotional insecurity. Chin J Clin Psychol. (2020) 28:1038–41+1037. 10.16128/j.cnki.1005-3611.2020.05.037

[23] Li ZH Zhu XW Tang ZJ Li T. Parent-child relationship and cyberbullying in adolescents: a moderated mediation model. Chin J Clin Psychol. (2020) 28:986–90. 10.16128/j.cnki.1005-3611.2020.05.02634777028

[24] Huang H Wang XY Lv B. Family cumulative risk and proble behavior of migrant preschool children: a moderated mediation model. Stud Psychol Behav. (2021) 19:779–85. 10.16187/j.cnki.issn1001-4918.2020.01.11

[25] Evans GW Li D Whipple SS. Cumulative risk and child development. Psychol Bull. (2013) 139:1342–96. 10.1037/a0031808 23566018

[26] Luo JJ Dong HN Ding QW Li DP. Cumulative ecological risk and adolescent internet addiction: a moderating role of effectful control. Chin J Clin Psychol. (2017) 25:893–96+901. 10.16128/j.cnki.1005-3611.2017.05.022

[27] Ge HY Liu AS. Cumulative familial risk index and adolescent self-harm behaviors. Chin J School Health. (2018) 39:698–701. 10.16835/j.cnki.1000-9817.2018.05.017

[28] Xiong JM Hai M Huang F Xin L Xu Y. Family cumulative risk and mental health in Chinese adolescents: the compensatory and moderating effects of psychological capital. Psychol Dev Educ. (2020) 36:94–102.

[29] Zheng MJ. Cumulative ecological risk in childhood and suicidal ldeation of college students with left-behind experience: a moderated mediating model. Chin J Special Educ. (2021) 11:74–82.

[30] Blum RW. A case for school connectedness. Educ Leadersh. (2005) 62:16–20. 10.1177/0013161X04269595

[31] Yu CF Liu S Wu T Zhang W. Parental corporal punishment and internet gaming disorder among adolescents: a moderated mediation model. J South China Norm Univ. (2017) 92–8+191.

[32] Guan J Sun Q. The effect of parent-child relationship on poverty children' problem behavior. J Psychol Sci. (2018) 41:1145–50. 10.16719/j.cnki.1671-6981.20180518

[33] Yuan YY Wang ZH Sun Q Wang DF Yin XY Li ZH. The relationship between cumulative family risk and emotional problems among impoverished children: a moderated mediation model. Psychol Dev Educ. (2022) 38:100–8. 10.16187/j.cnki.issn1001-4918.2022.01.12

[34] Hirschi T. Causes of Delinquency. Berkeley, CA: University of California Press (1969).

[35] Li JP Li DP Zhang W. The influence of parent-adolescent attachment and school connectedness on early adolescent's aggressive behaviors. China J Health Psychol. (2016) 24:68–72. 10.13342/j.cnki.cjhp.2016.01.016

[36] Xiao BS Wong YM. Cyber-bullying among university students: an empirical investigation from the social cognitive perspective. Int J Bus Inform. (2013) 8:34–69.

[37] Agnew RA. Foundation for a general strain theory of crime and delinquency. Criminology. (1992) 30:47–88. 10.1111/j.1745-9125.1992.tb01093.x

[38] Chu XW Fan CY Liu QQ Zhou ZK. Stability and change of bullying roles in the traditional and virtual contexts: a three-wave longitudinal study in Chinese early adolescents. J Youth Adolesc. (2018) 47:2384–400. 10.1007/s10964-018-0908-4 30171591

[39] Wu P Zhang Q Wangyang CZ. The relationship between parental style and cyber victimization of junior high school students: a longitudinal study. Psychol Dev Edu. (2021) 37:719–26. 10.16187/j.cnki.issn1001-4918.2021.05.13

[40] Jang H Song J Kim R. Does the offline bully-victimization influence cyberbullying behavior among youths? Application of General Strain theory. Comput Human Behav. (2014) 31:85–93. 10.1016/j.chb.2013.10.007

[41] Zhou HF Liu ZJ Fan YM Li BH. The mdiation of loneliness on the relationship between parent-child relationship and cyberbullying in junior high school students. Stud Psychol Behav. (2019) 17:787–94. 10.3969/j.issn.1672-0628.2019.06.010

[42] Huang H-W Chen J-L Wang R-H. Factors associated with peer victimization among adolescents in Taiwan. J Nurs Res. (2018) 26:52–9. 10.1097/JNR.0000000000000195 29315206

[43] Yin HW Jia LX. The research situation and development tendency of school bonding. J Psychol Sci. (2014) 37:1180–4. 10.16719/j.cnki.1671-6981.2014.05.03332971317

[44] Guo SY. A meta-analysis of the predictors of cyberbullying perpetration and victimization. Psychol Sch. (2016) 53:432–53. 10.1002/pits.2191424512111

[45] Gerard JM Buehler C. Cumulative environmental risk and youth maladjustment: the role of youth attributes. Child Dev. (2004) 75:1832–49. 10.1111/j.1467-8624.2004.00820.x 15566383

[46] Bao ZZ Li DP Zhang W Wang YH Sun WQ Zhao LY. Cumulative ecological risk and adolescents' academic and social competence: the compensatory and moderating effects of sense of responsibility to parents. Psychol Dev Educ. (2014) 30:482–95. 10.16187/j.cnki.issn1001-4918.2014.05.018

[47] Wadsworth ME Compas BE. Coping with family conflict and economic strain: The adolescent perspective. J Res Adolesc. (2002) 12:243–74.

[48] Furman W Buhrmester D. Children's perceptions of their personal relationships in their social networks. Dev Psychol. (1985) 21:1016–24. 10.1037/0012-1649.21.6.101625919481

[49] Dong Q Liu QQ. Introduction to the Standardized Test of Chinese Children and adolescents' Psychological Development. Beijing: China Science Publishing and Media Ltd (2011).

[50] Resnick M Bearman PS Blum RW Bauman KE Udry JR. Protecting adolescents from harmfindings from the national longitudinal study on adolescent health. JAMA. (1997) 278:823–32. 10.1001/jama.278.10.823 9293990

[51] Chen XZ Lai WP Ma HF Chen J Shan YT. The relation between parent-adolescent relationship and adolescent psychological capital: the mediating effect of friendship quality and the moderating effect of school bonding. Psychol Dev Educ. (2017) 33:544–53. 10.16187/j.cnki.issn1001-4918.2017.05.04

[52] Lam LT Li Y. The validation of the E-Victimisation Scale (E-VS) and the E-Bullying Scale (E-BS) for adolescent. Comput Human Behav. (2013) 29:3–7. 10.1016/j.chb.2012.06.021

[53] Ferrer-Cascales R Albaladejo-Blázquez N Sánchez-Sansegundo M Portilla-Tamarit I Lordan O Ruiz-Robledillo N. Effectiveness of the TEI program for bullying and cyberbullying reduction and school climate improvement. Int J Environ Res Public Health. (2019) 16:580–93. 10.3390/ijerph16040580 30781543 PMC6406958

[54] Hayes AF. Introduction to Mediation, Moderation, and Conditional Process Analysis: A Regression-Based Approach. New York, NY: Guilford (2013).

[55] Lian SL Sun XJ Zhou ZK Fan CY Niu GF. Social networking site addiction and undergraduate students' irrational procrastination: the mediating role of social networking site fatigue and the moderating role of effortful control. Plos ONE. (2018) 13:e0208162. 10.1371/journal.pone.0208162 30533013 PMC6289504

[56] Lian SL Sun XJ Niu GF Yang XJ Zhou ZK Yang C. Mobile phone addiction and psychological distress among Chinese adolescents: the mediating role of rumination and moderating role of the capacity to be alone. J Affect Disord. (2021) 279:701–10. 10.1016/j.jad.2020.10.005 33197839 PMC7539895

[57] Li DP Zhou YY Zhao LY Wang YH Sun WQ. Cumulative ecological risk and adolescent internet addiction: the mediating role of basic psychological need satisfaction and positive outcome expectancy. Acta Psychol Sin. (2016) 48:1519–37. 10.3724/SP.J.1041.2016.01519

[58] Li YJ. Effect of parent-adolescent conflict on cyberbullying: the chain mediating effect and its gender difference. Chin J Clin Psychol. (2020) 28:605–10+614. 10.16128/j.cnki.1005-3611.2020.03.035

[59] Patrick A Galassi JP. Gender and race as variables in psychosocial adjustment to middle and high school. J Educ Res. (2010) 98:102–8. 10.3200/JOER.98.2.102-108

[60] Bao ZZ Jiang YP Zhu JJ Zhang W. School connectedness and deviant peer affiliation among Chinese adolescents: the mediating role of sleep problems. Curr Psychol. (2020) 41:2152–61. 10.1007/s12144-020-00731-2

[61] Glassner SD Cho S. Bullying victimization, negative emotions, and substance use: utilizing general strain theory to examine the undesirable outcomes of childhood bullying victimization in adolescence and young adulthood. J Youth Stud. (2018) 21:1232–49. 10.1080/13676261.2018.1461200

[62] Kowalski RM Limber SP. Psychological, physical, and academic correlates of cyberbullying and traditional bullying. J Adolesc Health. (2013) 53:S13–20. 10.1016/j.jadohealth.2012.09.018 23790195

[63] Kim DH Lee JM Cho S Peguero AA Misuraca JA. From bullying victimization to delinquency in South Korean adolescents: exploring the pathways using a nationally representative sample. Child Youth Serv Rev. (2019) 98:305–11. 10.1016/j.childyouth.2019.01.018

[64] Cohen. Statistical Power Analysis for the Behavioral Sciences. New York, NY: Academic Press (1969).

[65] Zych I Baldry AC Farrington DP Llorent VJ. Are children involved in cyberbullying low on empathy? a systematic review and meta-analysis of research on empathy versus different cyberbullying roles. Aggress Violent Behav. (2018) 45:83–97. 10.1016/j.avb.2018.03.004

[66] Dishion TJ Nelson SE Bullock BM. Premature adolescent autonomy: parent disengagement and deviant peer process in the amplification of problem behaviour. J Adolesc. (2004) 27:515–30. 10.1016/j.adolescence.2004.06.005 15475044

[67] Zhang SL Li YH Dong JC. Research progress on family influencing factors of adolescent mental health. Sichuan Mental Health. (2015) 28:400–2.

[68] Logan-Greene P Nurius PS Hooven C Thompson EA. Life course associations between victimization and aggression: distinct and cumulative contributions. Child Adolesc Soc Work J. (2015) 32:269–79. 10.1007/s10560-014-0358-0 26190899 PMC4504828

[69] Su P Zhang W Yu CF Liu S Xu Y Zhen SJ. Influence of parental marital conflict on adolescent aggressive behavior via deviant peer affiliation: a moderated mediation model. J Psychol Sci. (2017) 40:1392–8.

[70] Podsakoff PM Mackenzie SB Podsakoff N. Sources of method bias in social science research and recommendations on how to control it. Soc Sci Electron Publ. (2012) 63:539–69. 10.1146/annurev-psych-120710-100452 21838546

